# Hepatic Angiosarcoma: An Unusual Case of Intractable Gastrointestinal Bleeding

**DOI:** 10.4021/gr539w

**Published:** 2013-05-03

**Authors:** Chijioke Enweluzo, Dipendra Chaudry, John Kennedy Sir Philip, Fahad Aziz

**Affiliations:** a Section on Hospital Medicine, Department of Internal Medicine, Wake Forest Baptist University Medical Center, Medical Center Boulevard, Winston Salem, NC 27101, USA; b Department of Pathology, Wake Forest Baptist University Medical Center, Medical Center Boulevard, Winston Salem, NC 27101, USA

**Keywords:** Angiosarcoma, Hepatic, Metastatic, Intractable gastrointestinal bleeding, CD31, CD34

## Abstract

Hepatic Angiosarcoma is an extremely rare malignant neoplasm of the vascular or lymphatic endothelium accounting for about 2% of all sarcomas. It is considered idiopathic in up to 70% of cases. We describe the case of a 32-year female transferred to our center for evaluation of intractable gastrointestinal bleeding. Definitive diagnosis remained elusive despite multiple endoscopic assessments with repeated cauterization of arteriovenous malformations (AVMs). Autopsy results confirmed metastatic hepatic Angiosarcoma.

## Introduction

Hepatic Angiosarcoma is an extremely rare malignant neoplasm of the vascular or lymphatic endothelium accounting for about 2% of all sarcomas [1]. Angiosarcoma is known to have a protean distribution affecting multiple organ systems including skin, soft-tissue, heart, great vessels, breast or liver. As expected, presenting features vary depending on the area of origin and metastatic sites. Bleeding is a characteristic presentation of this disease considering the vascular nature of the lesion. Our experience as highlighted in the case below shows that rare causes such as Angiosarcoma of any organ system should be extensively considered and investigated in any patient presenting with intractable gastrointestinal bleeding especially in the face of multiple negative endoscopic evaluations.

## Case Report

**Figure 1:**
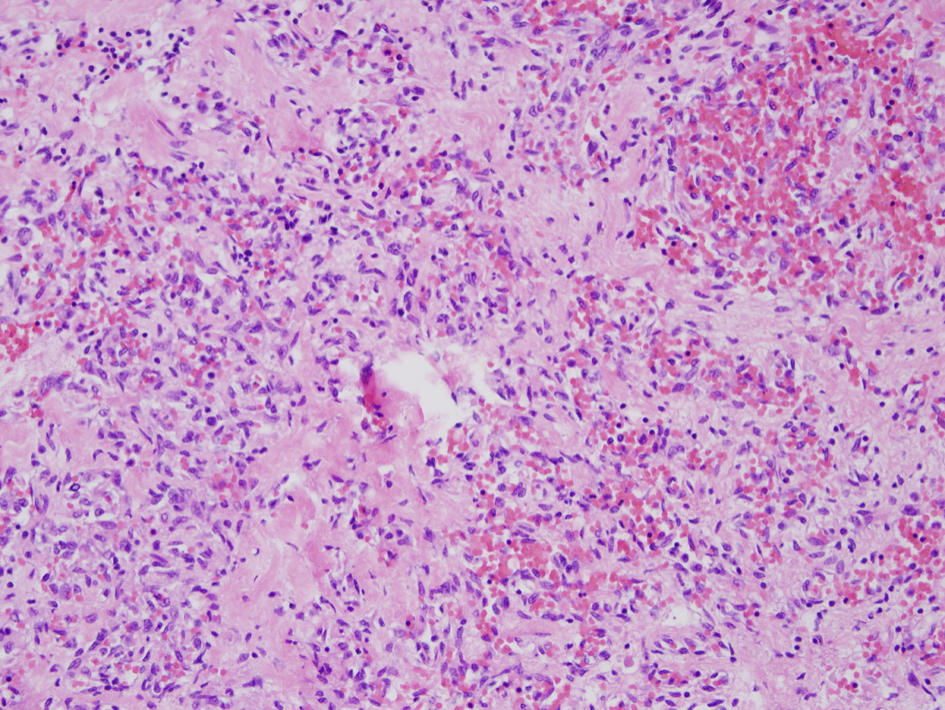
Sections from the lesion in the ill-defined scar in the right lobe of liver show markedly atypical endothelial cells forming irregularly proliferating vascular channels without appreciable vascular spaces. The tumor cells stain positive for CD31 and CD34 but negative for human herpes virus 8 (H&E × 200).

**Figure 2:**
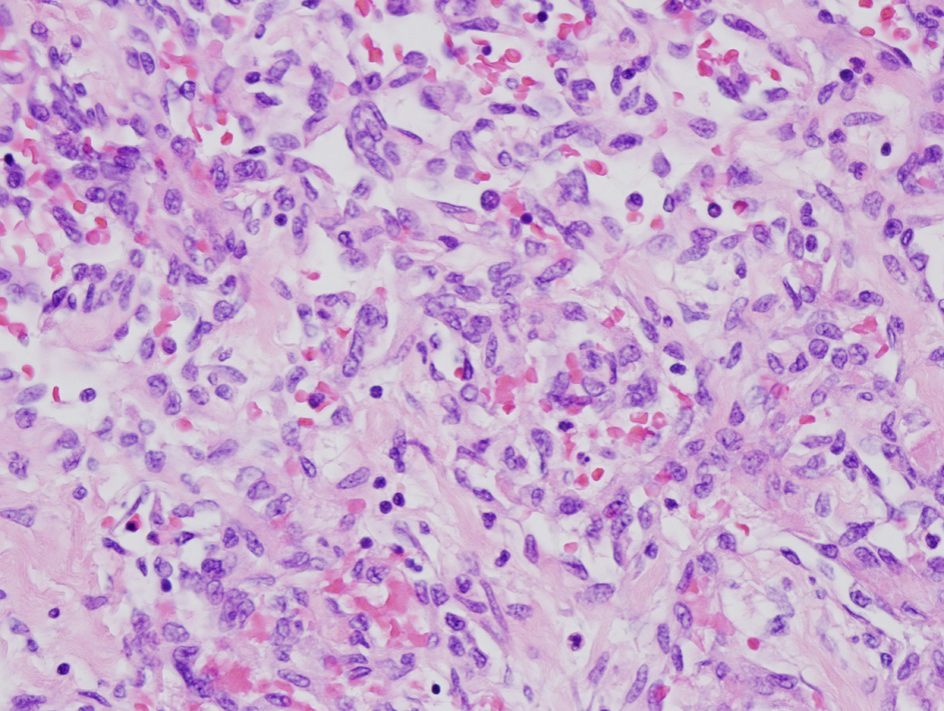
Sections from the lesion in the ill-defined scar in the right lobe of liver show markedly atypical endothelial cells forming irregularly proliferating vascular channels without appreciable vascular spaces. The tumor cells stain positive for CD31 and CD34 but negative for human herpes virus 8 (H&E × 400).

We describe the case of a 32-year-old female with a past medical history of anemia who was transferred to our center for further assessment and management of multiple medical problems including anemia, GIB, hemoptysis, and reportedly lung and liver lesions. She reported to the outside center with a 7-month history of progressive fatigue, hemoptysis, splinter hemorrhages and diffuse joint pain that involved both knees, hips, and left wrist. At the outside hospital her severe anemia persisted despite 9 units of PRBC transfusions. She underwent extensive assessment including esophagogastroduodenoscopy (EGD) and colonoscopy. EGD revealed gastric lesions consistent with AVMs while colonoscopy was negative. A bronchoscopy was negative but CT imaging showed liver and lung lesions of unknown etiology. On presentation to our center, vitals and physical examination were unremarkable. Initial blood work was remarkable for anemia, thrombocytopenia and hypokalemia. Repeat EGD and capsule endoscopy revealed active bleeding in the duodenum secondary to diffuse mucosal vascular lesions. Due to persistent bleeding she was transferred to the intermediate care unit (IMC) for closer monitoring. She continued to receive transfusions of PRBCs, FFP, cryoprecipitate, and platelets on a daily basis. During her hospitalization, she received a total of 4 EGD, 2 capsules and a push enteroscopy with similar findings of blood in the stomach, duodenum and small bowel with scattered AVMs, which were repeatedly cauterized. A bone marrow biopsy with pathology was inconclusive but concerning for possible angiosarcoma. A PET CT skull base to mid thigh did not show any evidence of malignancy but revealed diffuse low-level activity through out the axial skeleton that was deemed secondary to increased hematopoiesis. About 2 weeks following admission to our center, the patient became hypotensive from worsening hematemesis. She was transferred to the medical ICU. In the ICU, she continued to require daily transfusions of blood products with persisting GI bleed. She was started on estrogen/progesterone therapy, Avastin and aminocaproic acid for suspected hereditary hemorrhagic telangiectasia (HHT). Subsequently, she developed fulminant DIC and was rapidly transfused blood, factors, and platelets with improvement. A few days later, she developed respiratory failure requiring intubation and mechanical ventilation in addition to hemorrhagic shock requiring pressors and continued transfusions. Given her extremely poor prognosis, and through multiple discussions, the family decided against further escalation of care. Autopsy revealed angiosarcoma most likely arising from the liver and spreading to the lungs, spleen, mediastinal lymph node, gastrointestinal tract, skin and bone marrow. The liver appeared to be the primary site due to the size of the lesion involving approximately 75% of liver compared to the diffuse patchy involvement of lungs and spleen. Immunohistochemistry with CD31 and CD34 which are markers specific for endothelial cells and vascular tumors such as Angiosarcoma positively stained the tumor (Fig. 1, 2). The immunohistochemical stain human herpes virus 8 (HHV) did not stain the tumor making a diagnosis of Kaposi’s sarcoma unlikely. Gene sequence of ACVRL1, ENG and SMAD4 with deletion/duplication analyses of ACVRL1 and ENG specific for HHT did not detect any abnormality in an ante mortem whole blood sample and the vascular lesions evaluated were not AVMs.

## Discussion

Hepatic Angiosarcoma is an extremely rare malignant neoplasm of the vascular or lymphatic endothelium accounting for about 2% of all sarcomas [1]. It is considered idiopathic in up to 70% of cases but chronic exposure to thorotrast, vinyl chloride, arsenicals, radium and possibly copper have been implicated as causative factors [2, 3]. The highest incidence is between the sixth and seventh decade of life, youth and children is rare, predominantly in men compared to women (3:1). Angiosarcoma is known to have a protean distribution affecting multiple organ systems including skin, soft-tissue, heart, great vessels, breast or liver. As expected, presenting features vary depending on the area of origin and metastatic sites. When clinical manifestations begin, progression is often fast, and possibilities for curative treatment are limited [4]. Bleeding is a characteristic presentation of this disease considering the vascular nature of the lesion. The occurrence of thrombocytopenia and DIC may be related to local consumption of clotting factors and formed blood elements in the tumor. Localized, low-grade sarcomas are considered stage I, while large, high-grade, deep seated sarcomas are stage III. If a sarcoma does not have all three features (large, high-grade, and deep), it is defined as stage II. Of course, lesions with evidence of spread to distant organs and systems including lymph nodes are considered Stage IV. Primary hepatic angiosarcoma may appear as a solitary or multiple, hyper vascular lesions with heterogeneously early and progressive enhancement on CT and angiography [5, 6]. However, pathological examination remains the best approach for confirming primary hepatic angiosarcoma. Surgical resection is the primary mode of treatment. Radiation is often added to enhance tumor control. Angiosarcomas have a particular ability to recur near the site the tumor first started. The risk of recurrence also depends on the stage of disease at diagnosis. In terms of chemotherapy, Doxorubicin or Taxane based therapies have shown promising results, however, early diagnosis and surgical resection remains the best approach [7].

### Conclusion

We believe that the case outlined above shows that metastatic angiosarcoma of any organ system should be suspected and actively investigated in any case of intractable gastrointestinal bleeding. This could lead to early diagnosis and definitive treatment which in many cases is surgical resection.
